# Genome sequence and comparative genomic analysis of a clinically important strain CD11-4 of *Janibacter melonis* isolated from celiac disease patient

**DOI:** 10.1186/s13099-018-0229-x

**Published:** 2018-01-22

**Authors:** Atul Munish Chander, Rakesh Kochhar, Devinder Kumar Dhawan, Sanjay Kumar Bhadada, Shanmugam Mayilraj

**Affiliations:** 1 Department of Biophysics, Panjab University, Chandigarh, India; 2 Department of Gastroenterology, Postgraduate Institute of Medical Education and Research, Chandigarh, India; 3 Department of Endocrinology, Postgraduate Institute of Medical Education and Research, Chandigarh, India; 4 Microbial Type Culture Collection and Gene Bank (MTCC), CSIR-Institute of Microbial Technology, Chandigarh, 160036 India

**Keywords:** Genome sequencing, *Janibacter melonis*, Virulence, Clinical pathogen

## Abstract

**Background:**

*Janibacter melonis* and other member of this genus are known to cause bacteremia and serious clinical comorbidities, but there is no study reporting about pathogenicity attributes of *J. melonis. Janibacter terrae* is known to cause lethal infection. Reporting the genome of *J. melonis* CD11-4 and comparative genomics with other members of genus has provided some novel insights that can enable us to understand the mechanisms responsible for its pathogenicity in humans.

**Results:**

Comparative genomic analysis by Rapid Annotation Server and Technology revealed the presence of similar virulence determinant genes in both *J. terrae* NBRC 107853^T^ and *J. melonis* CD11-4. Like *J. terrae* NBRC 107853^T^, *J. melonis* CD11-4 contained two genes responsible for resistance against β-lactam class of antibiotics and two genes for resistance against fluoroquinolones. Interestingly, *J. melonis* CD11-4 contained a unique gene coding for multidrug resistance efflux pumps unlike all other members of this genus. It also contained two genes involved in Toxin-antitoxin Systems that were absent in *J. terrae* NBRC 107853^T^ but were present in some other members of genus.

**Conclusions:**

Genome annotations of *J. melonis* CD11-4 revealed that it contained similar or more virulence repertoire like *J. terrae* NBRC 107853^T^. Like other gut pathogens, *J. melonis* possesses key virulence determinant genes for antibiotic resistance, invasion, adhesion, biofilm formation, iron acquisition and to cope with stress response, thereby indicating that strain *J. melonis* CD11-4 could be a gut pathogen.

## Background

The genus *Janibacter* belongs to phylum *Actinobacteria* and family *Intrasporangiaceae* [1], that are non-spore forming, non-motile, aerobic, oxidase variable, and catalase-positive Gram-positive organisms. Colonies formed by these bacteria are smooth, circular, convex, and vary in color from white to yellow [2]. Strain CM2104^T^ was proposed as novel species *Janibacter melonis* in 2004, which was isolated from a spoiled oriental melon in Korea [3]. Elsayed et al. [4], for the first time isolated this microbe from blood (a clinical source) of a patient who presented with an acute onset of low-grade fever, right-sided facial swelling with pain, headache, and erythema after being bitten by an insect on his cheek. On the first day of illness, the insect stinger was completely removed by using a kitchen knife. Intravenous antibiotic therapy with cefazolin (2 g every 8 h) had improved the patient’s symptoms.

*Janibacter terrae,* another species of the genus, were reported recently to cause bacteremia in humans, and antibiotic treatments had improved the condition of 2 patients, while 2 other immune-compromised patients died due to infection [2]. In this study, for the first time, genome sequence of a clinical isolate belonging to the genus *Janibacter* is reported, which is isolated from duodenal mucosa of a celiac disease (CD) patient.

Earlier, we have reported that CD co-occurs with a number of diseases [5–8], whereas some other studies reported that microbes/infections modulate the disease presentations in CD [9–13]. By sequencing the genomes of microbes, we attempted to identify the genetic basis of pathogenicity, particularly microbial virulence and its probable role in CD [14, 15]. Infections play important in autoimmune diseases and CD [12, 16], but the prevalence and role of *J. melonis* in CD is not known so far; thus our work will highlights this organism as a probable pathogen. Therefore, infection caused due to *J. melonis* may need treatments to improve the clinical condition of the patients.

Reporting genome sequence and comparative genomics of *J. melonis* CD11-4 with other members of genus has provided some important insights. This report may enable us to understand the genetic mechanisms responsible for its pathogenicity in human diseases.

## Methods

### Bacterial strain culture and characterization

Strain CD11-4 was recovered from duodenal mucosa of a CD patient who was tTG IgA-antibody (Ab) positive (> 100 U/ml) presented with gastrointestinal symptoms including abdominal pain and painful defecation. It was proposed as strain CD11-4 of *J. melonis*. The tissue samples from duodenal mucosa were recovered during endoscopy at the Postgraduate Institute of Medical Education and Research, Chandigarh, India. The samples were used for characterisation of culturable microbes in the patient. After homogenizing in sterile phosphate saline (PBS), the samples were centrifuged at 4000 rpm for 2 min to remove debris. The supernatant was recovered and serially diluted with PBS and plated on to tryptic soy agar (TSA; HiMedia, India), incubated at 37 °C for 36 h. Single colonies appearing on the plate were picked and streaked on TSA plates that were further passaged at least two times to obtain pure colonies. Genomic DNA extraction and amplification were performed as described previously [17].

The strain designated as CD11-4 matched most of the phenotypic features of *J. melonis* CM2104^T^ and CM2110 [3]. Strain CD11-4 was identified as *J. melonis* by using 16S rRNA gene sequencing from the genomic DNA and was confirmed through an analysis of 16S rRNA gene retrieved from its whole genome sequence. Gene coding for 16S rRNA has shown that the strain CD11-4 belongs to the genus *Janibacter* and is most closely related to *J. melonis* CM2104^T^ (99.52% identity; 100% sequence completeness, 7 bases difference of a total 1446 bases) followed by *J. terrae* NBRC 107853^T^ (98.42% identity: 100% sequence completeness, 22 bases difference of a total 1447 bases), *J. anophelis* CCUG 49715^T^ (98.48% identity: 100% sequence completeness, 22 bases difference of a total 1444 bases) and *J. cremeus* HR08-44^T^ (97.99% identity: 100% sequence completeness, 29 bases difference of a total 1446 bases).

### Genome sequencing, assembly, and gene annotations and comparative genomic analysis

Genome of the strain was sequenced at C-CAMP (http://www.ccamp.res.in/) next-generation genomics facility, Bengaluru, India using an Illumina HiSeq 2 × 100 platform. Library preparation and sequencing were performed according to methods described previously [16]. The prepared libraries were quantified and then validated for quality by running an aliquot on High Sensitivity Bio analyser Chip, Agilent. De Novo assembly was performed with CLC Genomics Workbench (v8.5.1, CLCbio, Arhus, Denmark). During assembly, word size was set 45 and bubble size was 98. Default setting was used for read filtering and trimming, with a quality score of 0.05 and a maximum ambiguous nucleotides of 2. For operation “Discard reads below length”, the number was set to 15.

Genome annotation for the strain was performed by using Rapid Annotation Server and Technology (RAST) [17–20]. For comparative genomic analysis, genomes of *J. limosus* NBRC 16128^T^ (type species of genus), *J. terrae* NBRC 107853^T^, *Janibacter indicus* LMG 27493^T^, *Janibacter anophelis* NBRC 107843^T^, *Janibacter hoylei* PVAS-1^T^, *Janibacter corallicola* NBRC 107790^T^ and *Janibacter* spp. HTCC 2649 were retrieved from genome database of NCBI and were also annotated by using RAST.

As described previously [16], we identified unique genes, potential pathogenicity determinants, genes involved in metabolic pathways related to virulence, and common genes among three strains *J. limosus* NBRC 16128^T^ (reference species of the genus), *J. terrae* NBRC 107853 (another reference strain*),* and *J. melonis* CD11-4. *J. limosus* NBRC 16128^T^ is the type species of the genus and was considered for genomic analysis along with *J. melonis* CD11-4 so that the evolutionary trends could be compared for presence of new/unique genes. *J. terrae* is the pathogenic isolate reported from the genus *Janibacter,* and known to be lethal in immunocompromised individuals. Thus, another objective was to identify the common key pathogenic determinant genes in *J. melonis* CD11-4 and *J. terrae* NBRC 107853^T^. Therefore, only *J. limosus* NBRC 16128^T^ and *J. terrae* NBRC 107853^T^ were chosen for pathogenomic comparison with *J. melonis* CD11-4. Genes responsible for multidrug resistance efflux pumps and β-lactamase (cephalosporin) were reported as unique in *J. melonis* CD11-4 when comparative analysis was performed in three organisms. To confirm the uniqueness of these genes in the whole genus, rest of the genomes were searched for the above mentioned two genes by using RAST.

BLAST Ring Image Generator (BRIG) software was used to visualize the genome sequence similarity among strain CD11-4 and all other strains of the genus in the form of a map [21].

## Results and discussions

### Genome features

Draft genome of *J. melonis* strain CD11-4 consisted of 3,196,878 bp with G + C content of 73%. As per RAST annotations, it had 3064 coding sequences, 311 subsystems, and 49 total RNAs (Table 1). During assembly of the genome in CLC Genomics Workbench (v8.5.1, CLCbio, Arhus, Denmark), the strain was reported to have 12,007,098 reads. The data was preprocessed to trim and remove low quality sequences and finally a total of 11,823,074 high quality, vector filtered reads were employed for assembly. The assembled genome contained 7 contigs with N50 contig length of 483,445 bp and the largest contig assembled measured 957,763 bp.

**Table 1:** Genome features of *J. melonis* CD11-4

Genome annotations/features	*J. melonis* strain CD11-4
Accession no	LQZG00000000
Isolation source	Duodenal mucosa of CD patient
Size (Mb)	3.2
Contigs	7
G + C	73
tRNA	46
Other RNA	3
No. of RNAs	49
No. of subsystem	311
Proteins/coding genes	2936

### Comparative genomics

To calculate Average Nucleotide Identity (ANI), all the genomes were compared with strain CD11-4 by using ANI calculator [22, 23]. No other genome was available for *J. melonis* in the genome database of NCBI. When genome sequence of *J. melonis* CD11-4 was compared with *J. terrae,* ANI value had been reported 77.28%. The ANI value was 76.59% for *J. limosus*, 77.22% for *J. anophelis*, 76.57% for *J. corallicola*, 77.41% for *J. hoylei*, 77.46% for *J. indicus,* and 74.07% for *J.* spp. During the ANI calculations of *J. melonis* CD11-4 versus other genomes, the genome coverage was reported in a range of 26.02–38.03% for *J. melonis* and 19.67–37.39% for the compared genomes.

#### Virulence, disease and defense

By using RAST server, comparative genomic analysis was performed among *J. limosus* NBRC 16128^T^*, J. terrae* strain NBRC 107853^T^, and *J. melonis* CD11-4. As per classification of protein functions in RAST, category Virulence, disease and defense (VDD) consists of two main sub-categories that include (i) resistance to antibiotics and toxic compounds and (ii) invasion and intracellular resistance. Virulence determinant genes for resistance to antibiotics and toxic compounds have clinical significance, whereas genes belonging to the subcategory invasion and intracellular resistance are other possible cause of infection.

In category VDD, *J. terrae* NBRC 107853^T^ had 40 genes, whereas *J. melonis* CD11-4 had 36 genes, and *J. limosus* NBRC 16128^T^ had 34 genes. Subcategory “Invasion and intracellular resistance”, contains 15 genes in strains *J. melonis* CD11-4 and *J. terrae* NBRC 107853^T^ whereas 13 genes are in *J. limosus* NBRC 16128^T^. In subcategory, Resistance to antibiotics and toxic compounds, out of total 28 genes, *J. melonis* CD11-4 had 26 genes, *J. terrae* NBRC 107853^T^ had 25 genes and *J. limosus* NBRC 16128^T^ had 21 genes.

##### Resistance to antibiotics and toxic compounds

While comparing *J. terrae* NBRC 107853^T^ and *J. melonis* CD11-4, 4 genes were common in subsystem Arsenic resistance, 2 genes were common in subsystem β-lactamase, 3 genes were common in subsystem zinc–cadmium resistance, 4 genes were common in subsystem copper homeostasis, and 1 gene was common in subsystem resistance to vancomycin, 4 genes were common in subsystem resistance to fluoroquinolones, 1 gene was common in copper homeostasis, 1 gene was common in subsystem cadmium resistance and 2 genes were common in subsystem mercuric reductase. In subsystem β-lactamase, like both *J. terrae* NBRC 107853^T^ and *J. melonis* CD11-4, reference strain *J. limosus* NBRC 16128^T^ also had similar set of genes but genes coding for β-lactamase class C, other penicillin binding proteins and metal-dependent hydrolases of the β-lactamase superfamily III were absent in it.

**Fig. 1:**
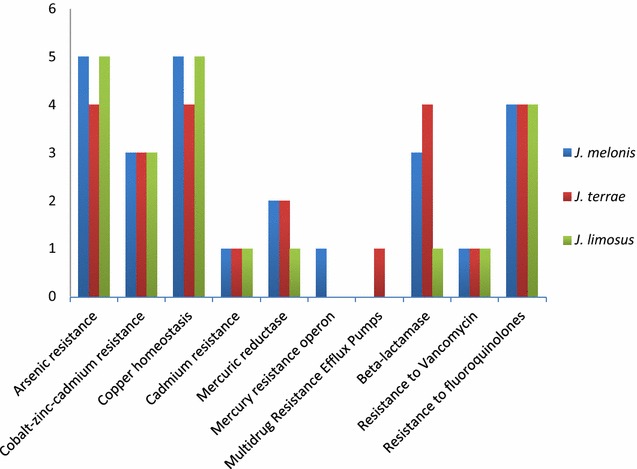
Genes responsible for antibiotic resistance and toxic compounds. The figure represents comparison about the numbers of genes present in various subsystems among three strains, *J. limosus* NBRC 16128^T^, *J. terrae* NBRC 107853^T^ and *J. melonis* CD11-4

One gene in each subsystem arsenic resistance, β-lactamase (cephalosporin), and multidrug resistance efflux pumps was unique in *J. melonis* CD11-4 that may make it a potent drug resistance strain in comparison to other strains of the genus. Interestingly, a unique subsystem, multidrug resistance efflux pumps, was present in *J. melonis* CD11-4 that contained a gene coding for multi antimicrobial extrusion protein (Na (+)/drug antiporter) belonging to MATE family of MDR efflux pumps (Fig. 1). We reported that this gene is absent in all other members of this genus conferring it’s uniqueness over the whole genus. The data supports clinical adaptabilities developed by strains *J. melonis* CD11-4 and *J. terrae* NBRC 107853^T^.

The strain CD11-4 had shown resistance only against nitrofurantoin (300 mcg) and sensitivity to all other antibiotics during antibiotic assay performed by using Icosa universal-2 and Icosa pseudo-2 panel (HiMedia, India). Icosa universal-2 panel assay revealed sensitivity of strain CD11-4 for antibiotics namely, amikacin, ampicillin, amoxycillin, cefadroxil, cefoperazone, ceftazidime, ceftriaxone, chlorampheniol, ciprofloxacin, cloxacillin, co-trimoxazole, erythromycin, gentamicin, nalidixic acid, netillin, nitrofurantoin, norfloxacin, penicillin, tobramycin, and vancomycin. When antibiotic assay was performed by using Icosa pseudo-2 panel, strain CD11-4 has shown sensitivity to other additional antibiotics gentamicin, imipenem, carbenicillin, piperacillin, aztreonam, ticracillin, levofloxacin, ticarcillin, colistin, cefepime, and piperacillin. The reason for showing sensitivity against most of the antibiotics may rely on the fact that *J. melonis* is less reported from clinical samples and therefore might have been less exposed to the antibiotics but having appropriate genetic machinery may develop antibiotic resistance in this microbe on exposure to various clinical environments in the future.

Looking at the pathogenic role of microbes of this genus, we first time report the antibiotic susceptibilities exhibited by *J. melonis* CD11-4 so that it’s further reports from clinical samples can be managed to improve patient’s health.

On the other hand, *J. terrae* NBRC 107853^T^ had one unique gene in subsystems cobalt–zinc–cadmium resistance, copper homeostasis, and mercury resistance operon that is not of clinical relevance. *J. limosus* NBRC 16128^T^ had two unique genes for copper homeostasis and a gene for resistance to chromium compounds. Genomic profile of antibiotic resistance is suggestive of it’s intestinal origin because food and human gut are reservoirs of antibiotic resistant genes [24, 25]. Thus, presence of several antibiotic resistance genes and unique genes in this category suggests the evolutionary transition of microorganisms into a gut pathogen.

##### Invasion and intracellular resistance

*Janibacter melonis* CD11-4 contains operon systems that are known for virulence and invasion in *Mycobacteria.* In this sub-category, *J. melonis* CD11-4 had same set of 15 genes like *J. terrae* NBRC 107853^T^. Two genes were present in subsystem *Mycobacterium* virulence operon involved in DNA transcription, 3 genes in *Mycobacterium* virulence operon involved in an unknown function with a Jag Protein and YidC and YidD, 3 genes in *Mycobacterium* virulence operon involved in protein synthesis (LSU ribosomal proteins), 4 genes in *Mycobacterium* virulence operon involved in protein synthesis (SSU ribosomal proteins), and 3 genes in subsystem *Mycobacterium* virulence operon possibly involved in quinolinate biosynthesis.

Two genes of subsystem *Mycobacterium* virulence operon involved in an unknown function with a jag protein and YidC and YidD were absent in *J. limosus* NBRC 16128^T^ that were otherwise common in other two strains. One of these genes coded for inner membrane protein translocase component YidC and the other gene coded for protein YidD.

##### Other virulence factor genes

Attributed to other potential virulence determinant genes, CD11-4 has some important genes that are also common in other members of the genus. In a subcategory, Programmed Cell Death and Toxin–Antitoxin Systems belonging to category, Regulation and Cell signaling, a gene coding for possible toxin to DivIC and another gene coding for a cell division protein DivIC (FtsB) that stabilizes FtsL against RasP cleavage was present. Such genes may activate under stress conditions to promote growth arrest, dormancy, and biofilm formation [28]. Interestingly, these genes were absent in *J. terrae* NBRC 107853^T^.

In category Regulation and Cell signaling, *J. melonis* CD11-4 had 2 genes in the subsystem Stringent Response for (p)ppGpp metabolism. These genes regulate the global expression and activity of many virulence regulators in response to the stress (nutrient starvation) exerted in host microenvironment. GTP pyrophosphokinase (p)ppGpp synthetase II and guanosine-3′,5′-bis(diphosphate) 3′-pyrophosphohydrolase are the two genes with above said roles that are also referred to as nucleotide alarmones due to their involvement in activating global signaling networks of virulence genes [29]. Like *J. melonis,* both the genes were also present in *J. terrae* NBRC 107853^T^ whereas *J. limosus* NBRC 16128^T^ lacked the gene coding for guanosine-3′,5′-bis(diphosphate) 3′-pyrophosphohydrolase. Most of the dreaded pathogens rely on nucleotide alarmones to counteract the stressful conditions arose due to immune responses, changes in nutrient supply or while adhering new surfaces [30].

In addition, utilization of sialic acid by *Vibrio cholera* (an enteric pathogen) promotes it’s colonization in gut [31]. Sialic acid utilization pathway is crucial for infection of *V. cholera* in human gut. We reported that *J. melonis* CD11-4 had genes coding for 7 enzymes that can metabolize sialic acids. These genes code for enzymes, glucosamine-1-phosphate *N*-acetyltransferase, glucosamine-fructose-6-phosphate aminotransferase, phosphoglucosamine mutase, *N*-acetylglucosamine-1-phosphate uridyltransferase, *N*-acetylglucosamine-6-phosphate deacetylase, UDP-*N*-acetylglucosamine 2-epimerase and sialic acid utilization regulator. Such genes were also present in *J. terrae* NBRC 107853^T^ but absent in *J. limosus* NBRC 16128^T^.

*Janibacter melonis* CD11-4 had 15 genes in subcategory oxidative stress. Out of these genes, 2 were coding for enzyme peroxidase and catalase to cope with the reactive oxygen species produced by human immune cells in an inflamed gut [26, 27]. Thus the presence of such genes may enable these microbes to escape from human immune cells around the leaky intestines in inflammatory diseases like CD [16]. Such genes are not only suggestive for the possible role of strain CD11-4 in CD but are also indicative to propose it as a gut pathogen [16]. Based on the above-mentioned genetic repertoire possessed by *J. melonis* CD11-4, it may be categorized as a gut pathogen.

In addition, inside the host, efficient uptake of iron is an important factor for survival of pathogens [32]. Several opportunistic pathogens possess such genes in their genomes [33]. Presence of potential genes for iron uptake may further add adaptability and virulence property in *J. melonis* CD11-4. *J. melonis* CD11-4 had 5 genes in category iron acquisition and metabolism. EfeUOB system of genes present in *J. melonis* CD11-4 are meant for high-affinity uptake of iron in *Bacillus subtilis* [34]. Two genes in the subsystem ABC transporters were also present that may promote ABC transporter-mediated uptake of iron. The genes of this category are also known to be responsible for inflammation and thus may have some role in pathogenesis of CD [16].

Infections are important factors in pathogenesis of CD and other autoimmune disorders. The above discussed genes in various gene categories Resistance to antibiotics and toxic compounds, Invasion and intracellular resistance, Oxidative stress, Iron acquisition system and Regulation and cell signaling are suggestive of virulent role of *J. melonis* CD11-4 in pathogenesis of CD.

### BRIG analysis

**Fig. 2:**
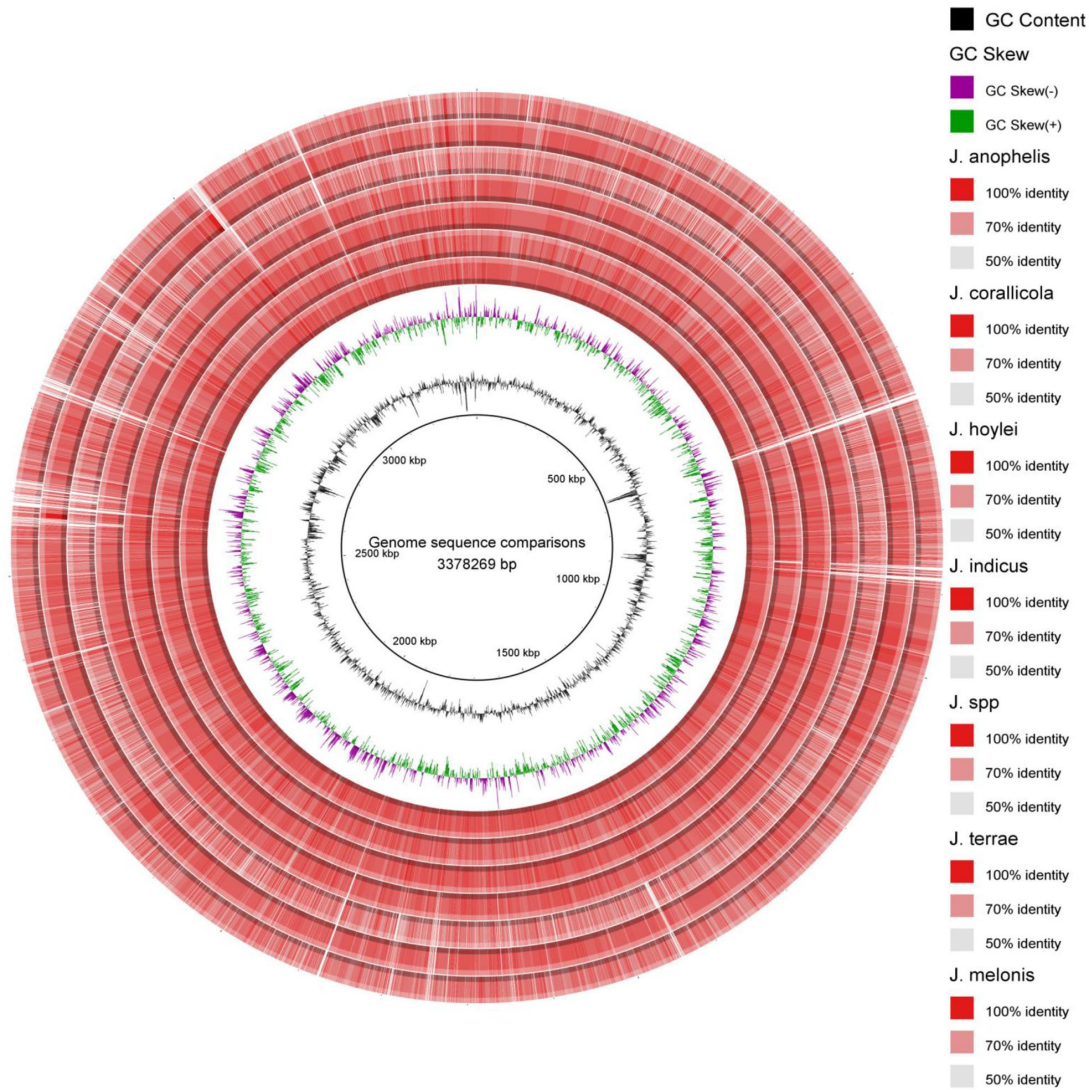
Whole genome sequence comparisons in microbes of genus *Janibacter*. The intensity of colour for each ring represents the BLAST match identity. Whole genome comparison of all the strains was conducted by considering *J. limosus* NBRC 16128^T^ as reference. Right side of the figure also represents sequence wise figure legends with an information on identity levels corresponding to the color match where the innermost circle (black circular line) represents reference genome (*J. limosus* NBRC 16128^T^), black irregular ring representing GC content, next outer ring represents GC skew, followed by that for *J. anophelis* NBRC 107843^T^, *J. corallicola* NBRC 107790^T^, *J. indicus* LMG 27493^T^, *J. hoyei* PVAS-1^T^, *J.* spp. HTCC 2649, *J. terrae* NBRC 107853^T^ and outermost ring is for strain *J. melonis* CD11-4

BRIG software, that works based on BLAST, was used to create a circular comparative map of whole genome. Darker areas of rings represent the 100% sequence similarity with the genome considered as reference (*J. limosus* NBRC 16128^T^), but the lighter (grey) areas represent 50% sequence similarity or less. Upper and lower thresholds for sequence identity were set as 70 and 50% subsequently. Genomes of *J.* spp. HTCC 2649 and *J. melonis* CD11-4 are showing maximum light bands representing more genetic variability while comparing with the reference genome of genus (*J. limosus* NBRC 16128^T^) (Fig. 2).

## Future directions

Considering the clinical importance of *J. melonis*, prevalence of these microbes need to be evaluated in the CD patients and in other inflammatory disorders of gastrointestinal track validated by respective disease models. Moreover, this report will highlight that the infections of *J. melonis* in humans may need to be cured for improving clinical condition of patients presenting gastrointestinal symptoms/diseases.

## Abbreviations

ANI: Average Nucleotide Identity
BRIG: BLAST Ring Image Generator
CD: celiac disease
PBS: phosphate saline
RAST: Rapid Annotation Server and Technology
